# A glucose-blue light AND gate-controlled chemi-optogenetic cell-implanted therapy for treating type-1 diabetes in mice

**DOI:** 10.3389/fbioe.2023.1052607

**Published:** 2023-02-10

**Authors:** Chi-Yu Li, Ting Wu, Xing-Jun Zhao, Cheng-Ping Yu, Zi-Xue Wang, Xiao-Fang Zhou, Shan-Ni Li, Jia-Da Li

**Affiliations:** ^1^ Furong Laboratory, Center for Medical Genetics, School of Life Sciences, Central South University, Changsha, China; ^2^ Hunan Key Laboratory of Animal Models for Human Diseases, Central South University, Changsha, China; ^3^ Hunan Key Laboratory of Medical Genetics, Central South University, Changsha, China; ^4^ Hunan International Scientific and Technological Cooperation Base of Animal Models for Human Disease, Changsha, China

**Keywords:** type-1 diabetes, optogenetic, chemigenetic, AND gate, cell-implanted therapy

## Abstract

Exogenous insulin therapy is the mainstay treatment for Type-1 diabetes (T1D) caused by insulin deficiency. A fine-tuned insulin supply system is important to maintain the glucose homeostasis. In this study, we present a designed cell system that produces insulin under an AND gate control, which is triggered only in the presence of both high glucose and blue light illumination. The glucose-sensitive GIP promoter induces the expression of GI-Gal4 protein, which forms a complex with LOV-VP16 in the presence of blue light. The GI-Gal4:LOV-VP16 complex then promotes the expression of UAS-promoter-driven insulin. We transfected these components into HEK293T cells, and demonstrated the insulin was secreted under the AND gate control. Furthermore, we showed the capacity of the engineered cells to improve the blood glucose homeostasis through implantation subcutaneously into Type-1 diabetes mice.

## Introduction

Diabetes mellitus is a metabolic disorder that affects more than 460 million people worldwide (Siehler et al., 2021). Although great progress has been made in diabetes research, the number of patients with diabetes is estimated to be 700 million by 2045 (Siehler et al., 2021). Approximately 10% of diabetic patients are Type-1 diabetes mellitus (T1D), which is one of the most common endocrinic and metabolic conditions occurring in childhood, and the symptoms can sometimes develop much later (Katsarou et al., 2017). T1D is characterized by complete or near-complete deficiency in insulin due to the loss of pancreatic islet β-cells (Katsarou et al., 2017; DiMeglio et al., 2018). The pathogenesis of T1D can be divided into three stages depending on the presence of hyperglycaemia and hyperglycaemia-associated symptoms (such as polyuria, thirst, hunger and weight loss) (Katsarou et al., 2017). By now, the mainstay therapy for T1D patients is exogenous insulin therapy, which is typically delivered *via* a subcutaneous insulin infusion or multiple daily injections (Katsarou et al., 2017; Beck et al., 2019; Warshauer et al., 2020). Novel therapeutic approaches with automatic supply of insulin is in demand (Siehler et al., 2021).

Replacement of lost β-cells with stem cell-derived β-like cells or engineered islet-like clusters is one of T1D therapies under investigation (Siehler et al., 2021). However, this technology is still immature. For instance, current engineering protocols for stem cell-derived β-like cells (SC-β-cells) produce few relevant cell types (e.g. SC-α-cells and SC-β-cells) involved in glucose control (Siehler et al., 2021). Further, SC-α-cells and SC-β-cells are also lost during sorting and reaggregation (Siehler et al., 2021). And the maturation of SC-β-cells to functional β-cells requires up to 1 year after transplantation (Robert et al., 2018).

Recently, a variety of synthetic biology-inspired approaches are developed to deliver therapeutic agents on demand by using trigger-controlled gene switches (Auslander and Fussenegger, 2013). Promising results have been generated in engineered non-islet cells that trigger the expression of gene of interest (GOI) (e.g., insulin) in response to glucose, electronic, light or non-canonical amino acids (Xie et al., 2016; Shao et al., 2017; Ye et al., 2017; Krawczyk et al., 2020; Wu et al., 2020; Li et al., 2021; Chen et al., 2022; Zhou et al., 2022). However, these single-trigger-controlled insulin expression systems remain risky in clinical application. For example, a light-triggered insulin synthesis system may be unexpectedly turned on by ambient light (Mansouri et al., 2021), whereas the glucose-triggered insulin synthesis system may fail to maintain high blood glucose at some specific conditions when the patients need a high blood glucose (e.g., during exercise) (Riddell et al., 2017).

In this study, we designed an AND gate-controlled cell to synthesize insulin under the control of both high glucose AND blue light stimulation. We termed it GBOI (glucose-blue light chemi-optogenetic cell-implanted therapy system). In this design, the insulin synthesis is controlled by an internal trigger of blood glucose and an external trigger of blue light (BL). This internal-external AND gate trigger-controlled insulin synthesis system ensures that insulin is synthesized only at the condition of both high blood glucose and BL stimulation. We further demonstrate the usefulness of the subcutaneously implanted GBOI-engineered cells for T1D treatment in mice.

## Materials and methods

### Molecular cloning and DNA constructs

The DNA clones used in this study, design of DNA constructs and the methods for molecular cloning are shown in Supplementary Table S1.

### Cell culture and transfection

Standard Dulbecco’s Modified Eagle’s Medium (DMEM; C11995500BT, Gibco) supplemented with 10% (vol/vol) fetal bovine serum (Certified Fetal Bovine Serum Functionally Tested for use with Tetracycline Regulated Systems; 04-005-1A, Biological Industries) and 1% (vol/vol) penicillin/streptomycin (15140-122, Gibco) was used for general cell culture. DMEM without glucose (11966-025, Gibco), supplemented with 10% (vol/vol) fetal bovine serum (04-005-1A, Biological Industries) and 1% (vol/vol) penicillin/streptomycin (15140-122, Gibco) was used for cell transfection. Cells were maintained at 37°C in a 5% CO_2_ atmosphere, and transfected with Lipo8000 Transfection Reagent (C0533FT, Beyotime) according to the manufacturer’s protocol. At 24 h after transfection, the cells were treated with blue light illumination as indicated. The cells were collected at 24 h after illumination for further assays.

### Enzyme-linked immunosorbent assay

To measure the insulin in the cell culture medium, cells were centrifuged for 10 min at 10,000 g. And the insulin concentration in the supernatant was determined by using an insulin enzyme-linked immunosorbent assay (ELISA) kit (DINS00, R&D Systems) according to the manufacturer’s protocol.

To determine the insulin concentration in serum, the mouse blood was allowed to clot for 30 min at room temperature. The samples were then centrifugated for 15 min at 1,000 g. And the insulin in the serum was determined using an insulin ELISA kit (DINS00, R&D Systems Inc.) according to the manufacturer’s protocol.

### Quantitative real-time PCR analysis

Total RNA was isolated from the cells using TRI Reagent Solution (AM9738, Invitrogen). A total of 1 μg of RNA was reverse-transcribed into cDNA using a PrimeScript RT Reagent kit (RR047, Takara). qPCR reactions were performed on the LightCycler 96 real-time PCR instrument (CFX96, Biorad) using Hieff UNICON^®^ Universal Blue qPCR SYBR Green Master Mix (11184ES08, Yeasen). The 2^−ΔΔCT^ method was used to calculate relative gene expression. The qPCR primers used in this study were listed in Supplementary Table S2.

### Dual luciferase reporter assay

Dual luciferase reporter assays were performed according to the manufacturer’s protocol (E2920, Promega). The signals of firefly luciferase (Luc) and renilla luciferase (Ren) were measured using the SIRIUS Luminometer (Berthold Detection Systems).

### Generation of microcapsule

A sodium alginate solution was prepared by dissolving 0.15 g sodium alginate in 15 mL cell culture medium. After stirring overnight at 4°C, the preparation was filtered through a 0.2 μm sterile syringe filter. Pelleted cells were mixed in the filtered sodium alginate solution to form a homogeneous suspension with a density of about 1.5 × 10^7^ cells per mL. The mixture was pumped through a 400 μm nozzle into a reaction chamber containing 100 mM CaCl_2_ cross-linking solution. The alginate microcapsule droplets were crosslinked in the CaCl_2_ solution for 5 min with gentle stirring, and then washed with PBS buffer.

### Construction of the type-1 diabetic mouse model

The T1D mouse model was generated as described previously (Shao et al., 2017). Briefly, 8-week-old male mice (C57BL/6) were fasted for 16 h and received intraperitoneal injection of streptozotocin (50 mg/kg body weight in 0.1 M citrate buffer, pH = 4) (S1312, Selleck) daily for consecutive 5 days. Two weeks after injection, mice with fasting glucose levels over 16 mM were considered as diabetic and used for further experiments. All procedures regarding the care and use of animals were approved by the Institutional Animal Care and Use Committee of Central South University of China.

### Blood glucose measurement

The glycemic profile of each mouse was monitored *via* tail vein blood samples using a glucometer (GA-6, Sinocare).

### Statistical analyses

Statistical significance was determined based on one-way ANOVA or Student’s t-test using SPSS 23.0 software (SPSS, USA).

## Results

### Rational design of the glucose-BL AND gate-controlled insulin synthesis system

GBOI was rationally designed to synthesize insulin under the internal glucose and the external BL AND gate control. GBOI includes the following components: 1) P_GIP_-GI-Gal4, a Gigantea (GI)-Gal4 fusion protein under the control of a GIP promoter (P_GIP_). 2) P_CMV_-LOV-VP16, a constitutively expressed LOV-VP16 fusion protein. 3) P_UAS_-Insulin, human pre-pro-insulin under the control of a tandem UAS promoter (P_UAS_).

The yeast transcription factor, Gal4, is able to drive gene expression under the UAS promoter (P_UAS_) (Giniger et al., 1985). The DNA binding domain of Gal4 was fused with GI (GI-Gal4), and the transactivation domain VP16 was fused to light oxygen voltage (LOV) domain of the plant photoreceptor FKF1 (LOV-VP16) (Yazawa et al., 2009). The GI and LOV domains interact following blue light activation, thus forming Gal4:VP16 complex to activate gene expression under the control of P_UAS_.

Glucose-dependent insulinotropic polypeptide (GIP) is a 42-amino acid gastrointestinal regulatory peptide that is induced by glucose (Boylan et al., 1997; Cheung et al., 2000). The promoter region of GIP is previously found to induce gene expression in a glucose-dependent manner (Boylan et al., 1997; Cheung et al., 2000), we thus chose it as a glucose-sensitive component in GBOI. The GIP promoter (P_GIP_, NM_004123.3) was inserted before the coding region for GI-Gal4 or firefly luciferase.

**FIGURE 1:**
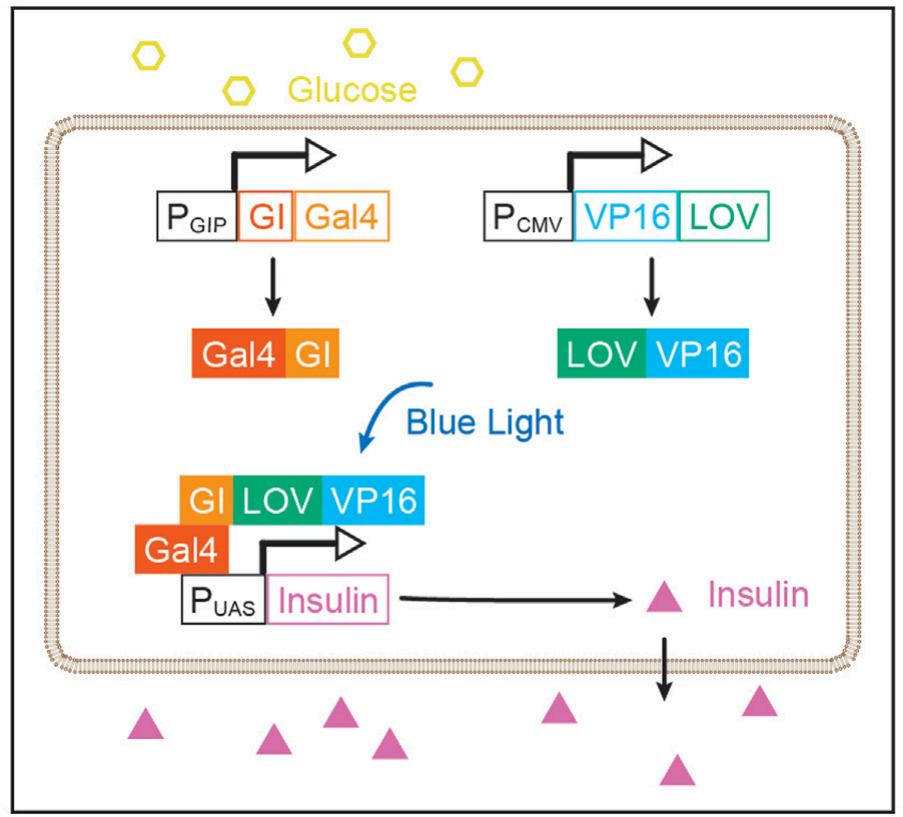
Rational design of GBOI system. A glucose-sensitive GIP promoter (P_GIP_) drives the expression GI-Gal4. The expression of LOV-VP16 fusion protein is under the control of a CMV promoter (P_CMV_). High glucose promotes the expression of GI-Gal4 through P_GIP_. And the blue light induces the interaction between GI and LOV, leading to the recombination of Gal4:VP16 transcriptional factor, which drives the insulin expression under the control of a tandem UAS promoter (P_UAS_).

The recombinant transcription factor Gal4:VP16 can induce gene expression under P_UAS_ control. Taking this advantage, we proposed to express insulin under the control of P_UAS_. In this study, the coding region of pre-pro-Insulin (CCDS_7729.1) was synthesized (Sangon Biotech). The pGL4.35 [luc2P/9XGAL4UAS/Hygro] vector containing nine repeats of UAS before the luciferase reporter gene (E137A, Promega) was used as the backbone to construct the P_UAS_-Insulin. The pre-pro-Insulin cDNA was inserted into the pGL4.35 between *Hind*III and *Fse*I restrictive enzyme sites to replace the luciferase gene (Figure 1).

Through the rational design of GBOI, we prospect to construct an AND gate-controlled insulin synthesis process in the cell. High glucose level will trigger the expression of GI-Gal4 through P_GIP_, and blue light illumination promotes the interaction of GI and LOV. Subsequently, the GI-Gal4:LOV-VP16 complex drives the expression of insulin under the P_UAS_. In this glucose-BL AND gate control module, the insulin will not be synthesized until both glucose and blue light are present (Figure 1).

### GBOI expresses insulin under glucose-BL AND gate control in cells

**FIGURE 2:**
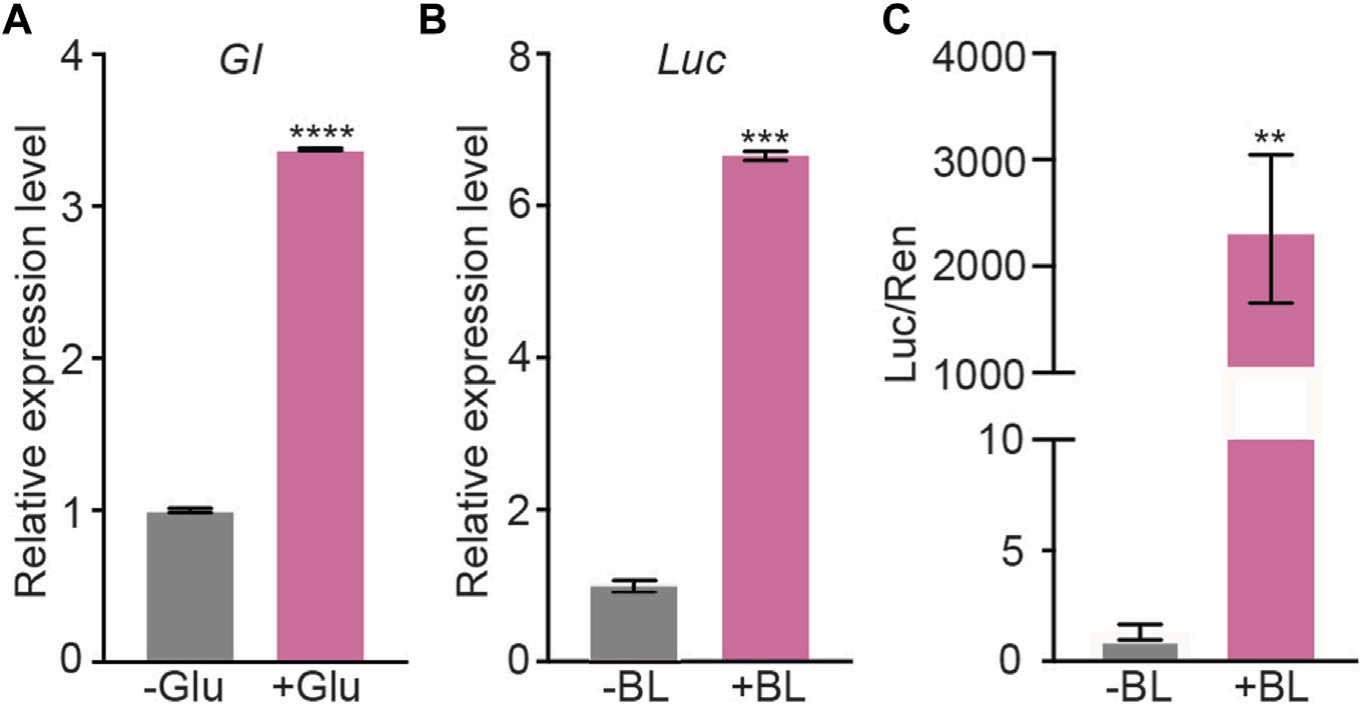
Validation of the glucose-sensitive trigger and blue light trigger in HEK293T cells. **(A)** The HEK293T cells were transfected with P_GIP_-GI-Gal4. 10 mM glucose (or vehicle as a negative control) was added into the cell culture medium to induce the gene expression. The expression level of *GI-Gal4* was detected by qRT-PCR. *Actin* was used as the reference gene. **(B,C)** P_CMV_-GI-Gal4, P_CMV_-LOV-VP16, P_UAS_-Luc and P_SV40_-Ren were co-transfected into HEK293T cells. The cells were cultured in presence of 10 mM glucose and illuminated by 1 μmol m^−2^ s^−1^ BL for 5 min (or kept in dark as the negative control) to induce the luciferase expression. The qRT-PCR **(B)** and dual-luciferase assays **(C)** were performed to detect the expression of luciferase. *Actin* was used as the reference gene in qRT-PCR assay. Data are presented as means ± SE (standard error). *n* = 2 in **(A,B)**; *n* = 6 in **(C)** *, *p* < 0.05, Student’s t-test. Data shown in this figure were performed in three biological replicates, and similar results were obtained.

To test the performance of GBOI, we first assessed the glucose-sensitivity of GIP promoter. The P_GIP_-GI-Gal4 plasmid was transfected into HEK293T cells, and the expression of GI-Gal4 was measured by using qRT-PCR (Figure 2A). As shown in Figure 2A, the mRNA level of GI-Gal4 was 3.4-fold higher in the presence of 10 mM glucose than that in the absence of glucose (Figure 2A), confirming glucose-dependent activity of GIP promoter.

Then, the transactivation of P_UAS_-Luc was used to assess the reconstitution of Gal4 DNA-binding domain and VP16 transactivation domain. P_CMV_-GI-Gal4, P_CMV_-LOV-VP16, and P_UAS_-Luc plasmids were transfected into the HEK293T cells (Figure 2), and the expression of luciferase was measured with qRT-PCR as well as luciferase activity. As shown in Figures 2B, C, blue light illumination induced 6.7-fold increase of the luciferase mRNA (Figure 2B) and more than 2,000-fold increase of the luciferase activity (Figure 2C). Both LOV-VP16 and GI-Gal4 are required for the induction of GOI expression under blue light illumination (Supplementary Figure S1). Red light was not able to induce the luciferase activity, indicating the specificity of blue light for the interaction of GI and LOV (Supplementary Figure S2). These results demonstrated the ability of BL to induce the interaction between GI and LOV and subsequent expression of P_UAS_-driven GOI.

**FIGURE 3:**
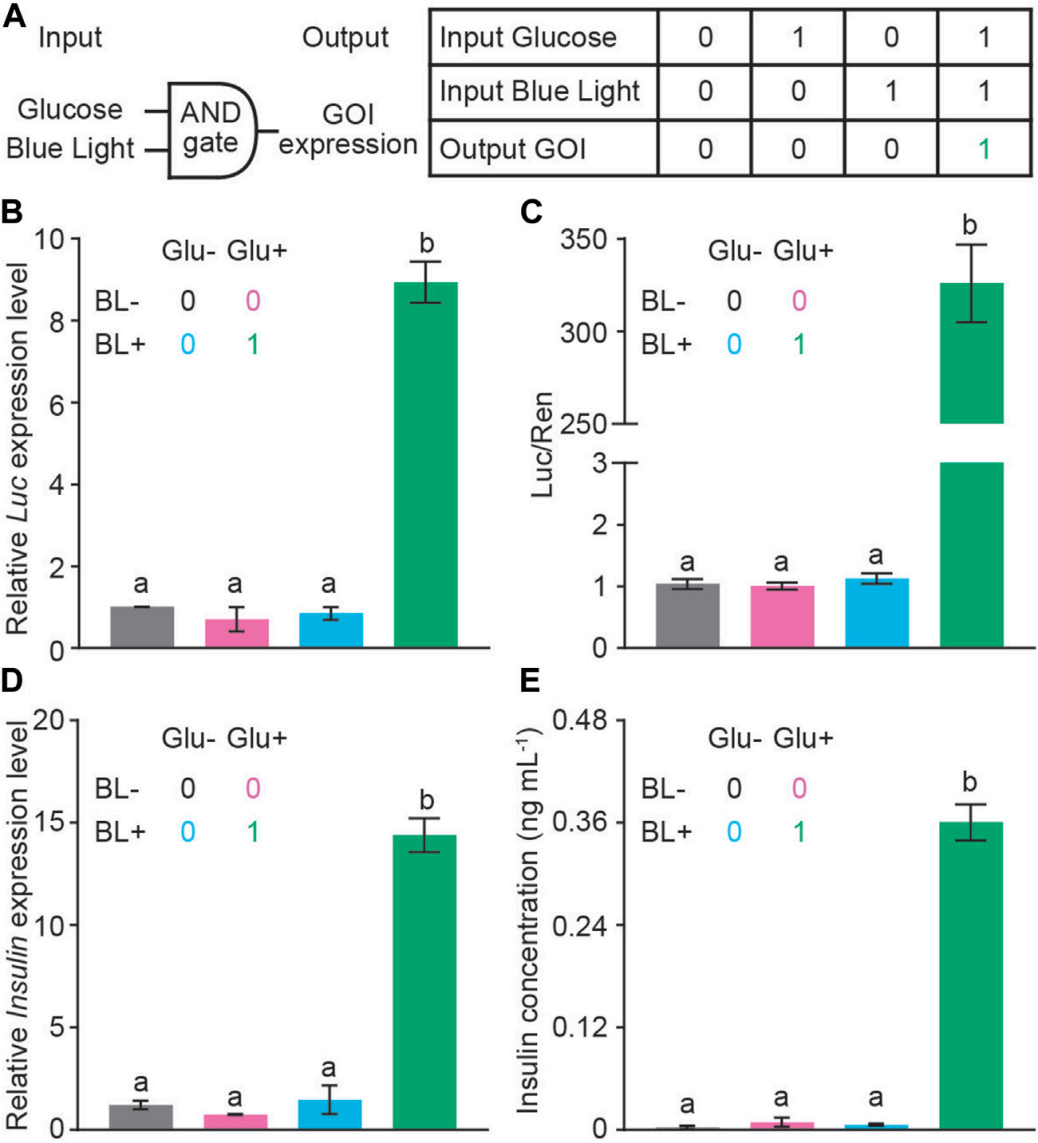
The ability of AND gate to control GOI expression. **(A)** A schematic diagram of an AND gate with the internal trigger of glucose and external trigger of BL (1 μmol m^−2^ s^−1^ for 5 min). Neither glucose nor blue light alone was able to turn on the insulin expression (Off status, indicated as the number 0). The GBOI system expresses GOI only when glucose and BL were presented simultaneously (On status, indicated as the number 1). **(B,C)** AND gate controls the expression of luciferase reporter. P_GIP_-GI-Gal4, P_CMV_-LOV-VP16, P_UAS_-Luc and P_SV40_-Ren constructs were co-transfected into HEK293T cells. The expression of luciferase was measured with qRT-PCR **(B)** or dual-luciferase assays **(C)**. **(D,E)** AND gate controls the expression of insulin as detected with qRT-PCR **(D)** and ELISA **(E)** assays. The colors of the bar are correlated to the Off or On status indicated as 0 or 1, respectively. *Actin* was used as the reference gene in **(B)** and **(D)**. Data are presented as means ± SE. *n* = 2 in **(A, C, and D)**; *n* = 5 in **(B)**. Values with different letters are significantly different (*p* < 0.05) from each other, using one-way ANOVA followed with Tukey’s post-hoc test. Data shown in this figure were performed in three biological replicates, and similar results were obtained.

Next, we evaluated the glucose-BL AND gate controlled GOI expression in HEK293T cells (Figure 3A). We tested the luciferase activity in response to 0 mM, 5 mM, 10 mM, 20 mM glucose, respectively. Five and 10 μM glucose steadily induced the luciferase activity; however, there was no significant difference in the luciferase activity under 10 mM and 20 mM glucose (Supplementary Figure S3). Therefore, 10 mM glucose was used as a high glucose level for the following studies. P_GIP_-GI-Gal4, P_CMV_-LOV-VP16 and P_UAS_-Luc were co-transfected into HEK293T, and a combinatorial approach was used to measure the expression of luciferase with qRT-PCR as well as luciferase activity (Figures 3B, C). As shown in Figures 3B, C, neither 10 mM glucose nor blue light illumination alone induced the expression of luciferase. However, the presence of both glucose (10 mM) and blue light induced 9-fold increase of the luciferase mRNA and 250-fold increase of the luciferase activity, respectively. These results thus in principle validated our glucose-BL AND gate design.

Finally, we replaced the luciferase coding region with the insulin expression cassette to see the glucose-BL AND gate control on insulin expression. As shown in Figures 3D, E, neither 10 mM glucose nor blue light illumination alone induced the expression of insulin in the qRT-PCR and ELISA assays. However, the presence of both glucose (10 mM) and blue light induced 14-fold and 300-fold increase in mRNA and protein levels of insulin, respectively. Thus, we have generated a designer cell to control insulin expression in the presence of high glucose and blue light AND gate.

### Therapeutic efficacy of GBOI in T1D mice

To study the performance of GBOI *in vivo*, we first tested whether BL could pass through the skin to control GBOI in the implanted cells (Supplementary Figures S4A–C). P_CMV_-GI-Gal4, P_CMV_-LOV-VP16 and P_UAS_-Luc were co-transfected into the HEK293T cells. The cells were covered by a piece of mouse skin and shined by BL (Supplementary Figures S4A, B). The luciferase expression was measured using qRT-PCR. As shown in Supplementary Figure S4C, the luciferase mRNA level was 34-fold higher under BL illumination than that kept in dark (Supplementary Figure S4C), indicating that BL could pass through the skin to trigger GBOI.

**FIGURE 4:**
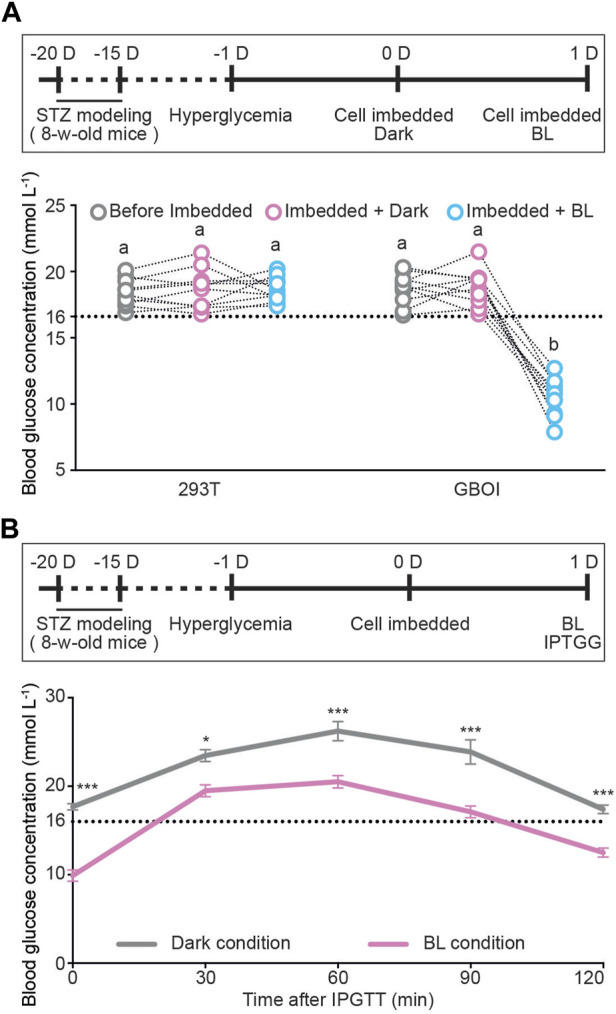
The ability of GBOI to ameliorate hyperglycemia in T1D mice. **(A)** The blood glucose levels of T1D mice implanted with HEK293T or GBOI-engineered HEK293T cells. Bars show the average blood glucose levels. Circles show the blood glucose levels of individual mouse. The experimental timeline is shown. 1 μmol m^−2^ s^−1^ blue light was applied for 15 min at the indicated time point. **(B)** Intraperitoneal glucose tolerance test (IPGTT) in the GBOI-engineered cells-implanted mice. The implanted mice received an intraperitoneal injection of glucose (1 g kg^−1^ body weight). After injection, the mice were shined by BL (1 μmol m^−2^ s^-1^) continuously (or kept in dark as the negative control). And the glucose levels of the mice were profiled from 0 to 120 min. The experiment timeline of IPGTT is shown. Data are presented as means ± SE. *n* = 10 in **(A,B)**. Values with different letters or * are significantly different (*p* < 0.05) from each other, using one-way ANOVA followed with Tukey’s post-hoc test. Data shown in **(A,B)** were performed in four and three biological replicates, respectively, and similar results were obtained.

Next, we tested the performance of GBOI in T1D mice. Eight-week-old C57BL/6 male mice were used to generate streptozotocin-induced T1D mice. Mice with fasting glucose levels over 16 mM were used for following experiments. HEK293T cells transfected with P_GIP_-GI-Gal4, P_CMV_-LOV-VP16 and P_UAS_-Insulin were alginate-microencapsulated and subcutaneously implanted onto the shaved dorsum of T1D-model mice (Supplementary Figure S4D). BL illumination was delivered by a LED array installed on the top of the cage (Supplementary Figure S4E), and the blood glucose and insulin levels were measured at 15 min after illumination. The blood glucose levels in the T1D mice implanted with HEK293T cells kept higher than 16 mM, no matter kept in dark or illuminated by BL (Figure 4A). In the GBOI-implanted T1D mice, the fasting blood glucose was still higher than 16 mM under dark condition (Figure 4A); however, 15 min BL illumination led to a significant drop of fasting blood glucose (Figure 4A).

In the GBOI-implanted mice, the insulin concentration is about 0.16 ng/mL without blue light illumination (Supplementary Figure S5); however, 15 min BL illumination led to a significant increase in the insulin concentration (∼0.73 ng/mL). Our results thus indicated that blue light induced insulin expression in GBOI-engineered cells to lower the glucose level in T1D mice (Supplementary Figure S5).

We also performed an intraperitoneal glucose tolerance test (IPGTT) on T1D mice implanted with the GBOI-engineered cells. After challenged with exogenous glucose (1 g kg^-1^ body weight), mice were continuously shined for 120 min by BL or kept in dark, and the glucose levels of the mice were profiled from 0 to 120 min. The BL shined GBOI-implanted mice had a significantly lower postpandial glucose level than the mice kept in dark (Figure 4B). The BL shined GBOI-implanted mice had an average of 12.5 mM blood glucose level at 120 min after challenge, whereas an average of 17.4 mM blood glucose level was found in the GBOI-implanted mice kept in dark (Figure 4B).

## Discussion

In this study, we designed a chemi-optogenetic AND gate trigger system to control insulin synthesis to treat T1D, termed GBOI (Figure 1). GBOI is consisted with an internal glucose-sensitive trigger, an external BL illumination trigger and a UAS promoter driven insulin expression cassette. Ideally, insulin will not be expressed unless the cells are stimulated by both high glucose and BL. We first confirmed that both glucose and BL triggers worked well in inducing GI-Gal4 expression and the interaction between LOV-VP16 and GI-Gal4, respectively in cells (Figure 2). Then the two separate modules were successfully assembled as an AND gate trigger to control the GOI expression in the cells (Figure 3). Our results demonstrated that the GBOI triggers GOI (e.g., insulin) expression only when the cells grown under high glucose content together with BL stimulation (Figure 3). We further demonstrated the ability of GBOI to lower glucose levels in T1D mice (Figure 4). GBOI system provides a potential solution for the dilemma of ambient light prevention and T1D patients’ temporary high blood glucose requirement.

Nowadays, optogenetics tools are widely used in precisely manipulating specific biology processes (Frank et al., 2018; Paoletti et al., 2019). GBOI offers an alternative for T1D patients to improve blood glucose homeostasis. First, the environmental ambient light may cause the unexpected trigger of light-only-controlled insulin synthesis. However, light will not turn on the GBOI system if the blood glucose is low. Second, insulin injection may keep blood glucose in T1D patients at a relative low level, which is not sufficient for some circumstances, such as exercising. Currently, T1D patients may intake some carbohydrate before sports to avoid the sport-induced hypoglycemia. In this scenario, the GBOI system could be deactivated manually, by turning off the blue light, to keep a high glucose supply.

Recently, a variety of non-invasive glucose monitors have been developed to detect the blood glucose in real time (Alsunaidi et al., 2021; Johnston et al., 2021; Xue et al., 2022). In principle, the non-invasive glucose monitors may be programed to control a blue light source. For instance, the blue light is turned on when the blood glucose level is over a set value, whereas the blue light is turned off when the blood glucose level falls below the threshold. We speculate that integration of such wearable medical devices with GBOI described in this study will offer a more precise control of blood glucose in T1D patients.

Naturally, insulin is stored in β-cells at a readily releasable status in the insulin secretory granules localized to the plasma membrane (Gaisano, 2017). The secretion of insulin involves a “triggering” pathway, which releases insulin with a sharp peak in short time, and an “amplifying” pathway, which releases insulin at a lower but sustained rate (Henquin, 2009). This store-release strategy allows β-cells response to high glucose signal and release insulin timely. However, the insulin production in most designer cells is regulated at the transcriptional level. In contrast to the quick rise of insulin in response to a glucose surge in native β-cells, it may take more time to synthesize and secret insulin proteins after the increase of blood glucose. Although such designer cells are able to normalize glucose homeostasis in T1D mouse models, an ideal β-cell-mimetic designer cell is expected to control insulin protein translation (Chen et al., 2022) or secretion.

## Data Availability

The raw data supporting the conclusion of this article will be made available by the authors, without undue reservation.

## Supplementary material

SUPPLEMENTARY FIGURE S1 The ability of light-responsive components to control GOI expression. HEK293T cells transfected with different combination of plasmids were illuminated by 1 μmol m^-2^ s^-1^ blue light for 5 min. And the expression of luciferase was measured with a dual luciferase assay. Neither LOV-VP16 nor GI-Gal4 alone was able to increase the luciferase activity. Only co-transfection of LOV-VP16 and GI-Gal4 led to increase in the luciferase activity. The colors of the bars are correlated to the four combinations of assays indicated in the legend, respectively. Data are presented as means ± SE. *n* = 5. Values with different letters are significantly different (*P* < 0.05) from each other, using one-way ANOVA with Tukey’s post-hoc test. Data shown in this figure were performed in three biological replicates, and similar results were obtained.

SUPPLEMENRTARY FIGURE S2 Blue light (BL, 1 μmol m^−2^ s^−1^ for 5 min), but not red light (RL, 1 μmol m^−2^ s^−1^ for 5 min), triggers the interaction between LOV and GI. P_CMV_-GI-Gal4, P_CMV_-LOV-VP16, P_UAS_-Luc and P_SV40_-Ren were co-transfected into HEK293T cells. The cells were cultured in the presence of 10 mM glucose and kept in dark (Dark) or illuminated by red light (RL) or blue light (BL), respectively. A dual luciferase assay was performed to detect the expression of luciferase. Data are presented as means ± SE. *n* = 6. ****, *P* < 0.0001, Student’s t-test. Data shown in this figure were performed in three biological replicates, and similar results were obtained.

SUPPLEMENTARY FIGURE S3 Glucose dose-dependently activates P_GIP_. P_GIP_-GI-Gal4, P_CMV_-LOV-VP16, P_UAS_-Luc and P_SV40_-Ren were co-transfected into HEK293T cells. After transfection, cells were cultured in the medium supplemented with different concentrations of glucose. The expression of luciferase was measured with a dual luciferase assay after 5 min of 1 μmol m^−2^ s^−1^ blue light illumination. Data are presented as means ± SE, *n* = 6. Values with different letters are significantly different (*P* < 0.05) from each other, using one-way ANOVA with Tukey’s post-hoc test. Data shown in this figure were performed in three biological replicates, and similar results were obtained.

SUPPLEMENTARY FIGURE S4 The cell-implanted mice were shaved and illuminated by BL. () penetration of the BL light through the mouse skin. (**C**) BL induces luciferase expression in HEK293T cells covered with a piece of mouse skin. P_CMV_-GI-Gal4, P_CMV_-LOV-VP16, P_UAS_-Luc were co-transfected into the HEK293T cells. The cells were covered with a piece of mouse skin and shined by BL (1 μmol m^−2^ s^−1^ for 5 min) (or kept in dark as the negative control). The expression levels of luciferase were measured with qRT-PCR. *Actin* was used as the reference gene. Data are presented as means ± SE. *n* = 2 in panel B. *, *P* < 0.05, Student’s t-test. Three biological replicates were performed, and similar results were obtained. (**D**) The mice were shaved to expose the skin. (**E**) A photo showing BL-shined mice.

SUPPLEMENTARY FIGURE S5 Blue light induces insulin synthesis in GBOI-implanted mice. GBOI-engineered HEK293T cells were implanted into T1D mice. The mice were illuminated by 1 μmol m^−2^ s^−1^ blue light for 15 min. And the insulin concentration is measured using an ELISA assay. Data are presented as means ± SE. *n* = 10 mice. ****, *P* < 0.0001, Student’s t-test. Data shown was performed in three biological replicates, and similar results were obtained.
