# Supplementary figures and images for: A glucose-blue light AND gate-controlled chemi-optogenetic cell-implanted therapy for treating type-1 diabetes in mice
Source: Front Bioeng Biotechnol. 2023 Feb 10;11:1052607. doi: 10.3389/fbioe.2023.1052607 (PMC9954140; doi:10.3389/fbioe.2023.1052607)

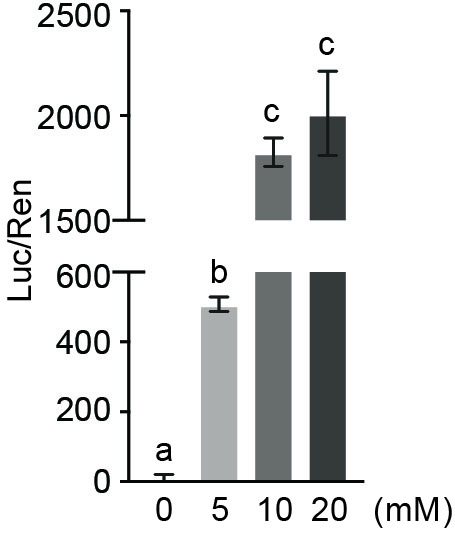

Supplement: Supplementary file 2 [file Image3.JPEG]

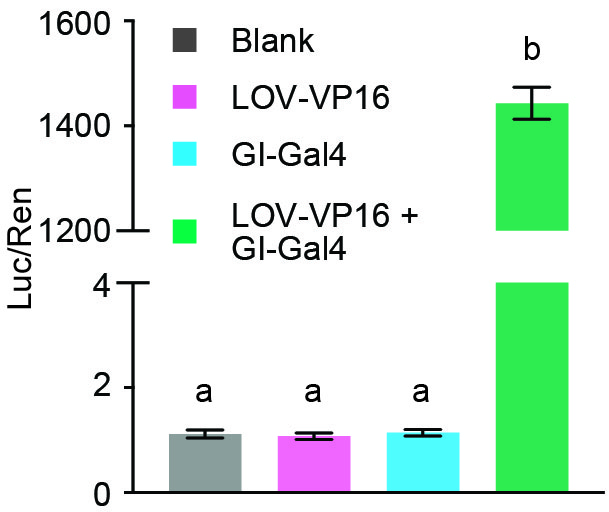

Supplement: Supplementary file 4 [file Image1.JPEG]

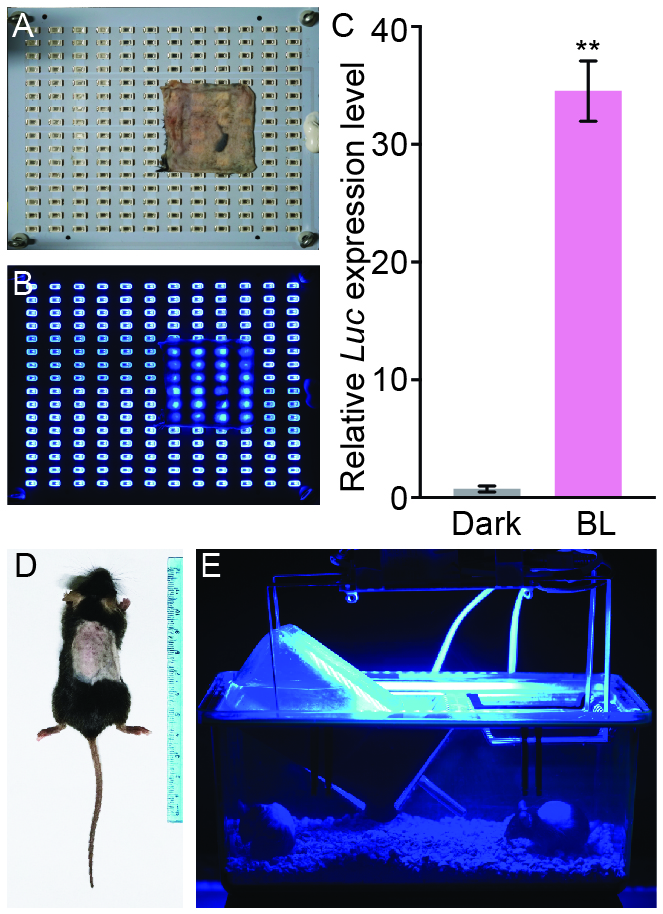

Supplement: Supplementary file 5 [file Image4.JPEG]

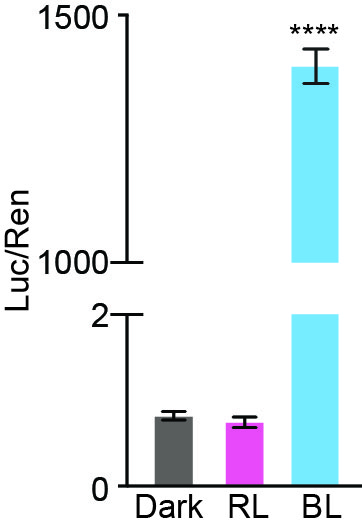

Supplement: Supplementary file 6 [file Image2.JPEG]

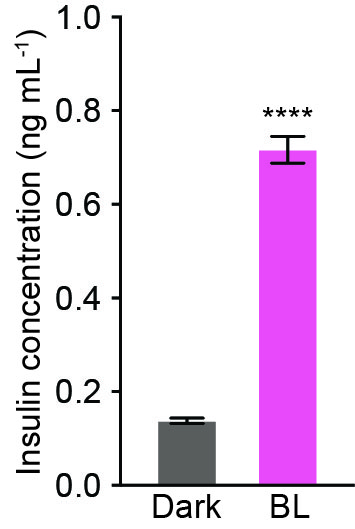

Supplement: Supplementary file 7 [file Image5.JPEG]
