# Supplementary material for: A glucose-blue light AND gate-controlled chemi-optogenetic cell-implanted therapy for treating type-1 diabetes in mice
Source: Front Bioeng Biotechnol. 2023 Feb 10;11:1052607. doi: 10.3389/fbioe.2023.1052607 (PMC9954140; doi:10.3389/fbioe.2023.1052607)
Supplement: Supplementary file 3 [file Table1.DOCX]

**Molecular cloning and DNA constructs**

1. P_GIP_ (NM_004123.3)

2. pre-pro-Insulin

CDS sequence (CCDS_7729.1):

atggccctgtggatgcgcctcctgcccctgctggcgctgctggccctctggggacctgacccagccgcagcctttgtgaaccaacacctgtgcggctcacacctggtggaagctctctacctagtgtgcggggaacgaggcttcttctacacacccaagacccgccgggaggcagaggacctgcaggtggggcaggtggagctgggcgggggccctggtgcaggcagcctgcagcccttggccctggaggggtccctgcagaagcgtggcattgtggaacaatgctgtaccagcatctgctccctctaccagctggagaactactgcaactag

Protein sequence:

MALWMRLLPLLALLALWGPDPAAAFVNQHLCGSHLVEALYLVCGERGFFYTPKTRREAEDLQVGQVELGGGPGAGSLQPLALEGSLQKRGIVEQCCTSICSLYQLENYCN

3. P_CMV_-GI-Gal4 (Addgene #42500)

4. P_GIP_-GI-Gal4

The promoter of GIP (NM_004123.3) was inserted in to the PCMV-GI-Gal4 (Addgene #42500) between the restriction enzymes of *Mlu*I and *Eco*RI.

Sequence:

atctctccagtcccttcctcaaccttctgagaacaggcaaactccaccatgattggcttataaatcgttatatggacctactaaggatgtaacaactgggagcatgcttacctagcatgtccgaaacccggagttcagtccctagcactgcacaatctcagtccttatgaagtagagggaagatcagaggttcaaggacaacatcaatttgagaccagcctgggctacttaccaaagaaagaaagagagaaataaataaatagatagataaataaataaataagtaaataaatatcttatggctggagagttggttcagtgtttaagagcacttattgtggggttggggatttatctcagtggtagagcgtttgcctaggaagctcaaggccctgggttcggtccccagctccggaaacaaaacaaaacaaaacaaaaacaaacaaacaaacaaaaaaccctgtctggaaaacacctaaataaagatatatatatataatatatatacatataatatatatatgatatatatatatatatatatctttgtggaggaagctatacctttctttcttgagcctccaacacataaatgtgccctgtcatcccattcatattgccccaagtgggaaaccatgtgactataaactctaagttcctagtcactaggaactctcaagacacctacctcaggcagcatcacttccggagtgccaccattatcagttaacatccacatctgggattcagatcccagatcccttctgttccctcagaagtcacctacagctttgtgggggtgccccttccctcagagagtgccacccgagttgaccctcaccaaggcaaccctttgtacccacagaatccaacaggaagtagggggaagaacagccggccctgtgcccagaaaaaaagaggggagggagaagggggtgctcagcctaccaccgggcaggtcccagataacactgcagatacccaaatgttaatcacccattagcacaggcccagagcaaaggggaaagtgattaggtgtataatggggttcactgggcaggagcagtgggcttgagcttcaaagataagaggttttcaggttaatcagcaccctgtggtgtgtggatataaggaagctaacacagggtcttgagaattcgccaccatggctagttcatcttcatctgagagatggatcgatggtcttcagttctcttccttgttatggcctccgccacgagatcctcaacaacataaggatcaagtcgttgcttatgttgaatattttggtcaatttacatcagagcaattcccagatgacattgctgagttggtccggcatcagtatccatcaaccgagaagcgacttttggacgatgtgctggcgatgtttgtccttcatcatccggagcatggtcatgcagtcattcttccaatcatttcatgtcttattgatggctcgttggtgtacagcaaggaagctcatccgtttgcctctttcatatctttagtttgcccaagtagtgagaatgactattcggagcaatgggctttggcatgtggagaaatccttcgcattttgactcattacaaccgtcccatttataaaactgagcagcaaaatggagatacagagagaaattgtctgagcaaagctacaactagtggttctccgacttcagagcctaaggctggatcaccaacacagcatgaaaggaaacctttaaggcctttgtctccatggatcagtgatatactacttgctgctcctcttggtataagaagtgactatttccgatggtgtagtggtgtaatgggtaaatatgctgctggagagctcaagccgccaaccattgcttctcgaggatctggtaaacatcctcaactgatgccttcaaccccaagatgggctgttgctaatggagctggtgtcatactgagtgtttgtgatgatgaagttgctcgatatgagactgctacgctgacagcggtcgctgtccctgcacttcttcttcctccgccaacgacatccttagatgagcatctagttgctggccttccagctcttgaaccatatgcacgtttgtttcatagatactatgccattgcaactccaagtgctacgcagagacttcttcttggactcttagaagcaccaccgtcgtgggctccagatgcacttgatgctgctgtacagcttgtggaactccttcgagctgctgaagattatgcatctggtgtaaggctacccaggaactggatgcatttgcacttcttgcgggctataggaattgctatgtctatgagggcaggtgttgctgctgatgctgcagccgctttgcttttccgcatactctcacagccggcactgctttttcctccgctaagtcaagttgagggagtagaaattcagcacgcgcctattggtggctacagttcaaattacagaaaacagatagaagttcctgcagcagaagcaaccattgaagccactgcccaaggaattgcctcaatgctttgtgctcatggtcctgaagttgagtggagaatttgcactatatgggaagctgcttatggtttgatccctttaaattcttcggcggttgatcttcccgaaatcatagttgctaccccactgcaacctcctatcttgtcatggaatttatacattccactcctcaaagtacttgaatatcttccacgggggagtccttcggaagcatgcttgatgaaaatatttgttgccactgtggaaacaatactcagtagaacttttccgcctgaatcttccagggaactaaccagaaaagctagatcgagttttaccacaagatcagcgaccaaaaatcttgctatgtctgagcttcgtgctatggtccatgctctctttttagaatcatgcgctggtgtggaattagcttcacgcctactttttgttgtgttgactgtatgtgttagccatgaagcacagtctagtggtagcaagagaccgagaagtgaatatgctagtactactgaaaatattgaggcgaatcaacctgtatctaacaatcaaactgctaaccgtaaaagtaggaatgtcaagggacagggacctgtggcagcatttgattcatacgttcttgctgctgtttgtgctcttgcctgtgaggttcagctgtatcctatgatctctggtggggggaacttttccaattctgccgtggctggaactattacaaagcctgtaaagataaatgggtcatctaaagagtatggagctgggattgactcggcaattagtcatacgcgccgaattttggcaatcctagaggcactcttttcattaaaaccatcttctgtggggactccatggagttacagttctagtgagatagttgctgcggccatggttgcagctcatatttccgaactgttcagacgttcaaaggccttgacgcatgcattgtctgggttgatgagatgtaagtgggataaggaaattcataaaagagcatcatcattatataacctcatagatgttcacagcaaagttgttgcctccattgttgacaaagctgaacccttggaagcctaccttaagaatacaccggttcagaaggattctgtgacctgtttaaactggaaacaagagaacacatgtgcaagcaccacatgctttgatacagcggtgacatccgcctcaaggactgaaatgaatccaagaggaaaccataagtatgctagacattcagatgaaggctcaggaagaccctcagagaagggtatcaaagatttcctcttggatgcttctgatctagcgaatttcctcacagctgatagactcgcagggttctattgtggtacacaaaagcttttgaggtcagtgcttgcagagaaaccggagctgtctttctccgttgtttcactgttatggcacaaactgattgctgctcctgaaatccagcccaccgcagaaagcacctctgcgcaacaaggatggagacaggttgttgatgcgctatgcaatgtcgtatctgcaacgccagcgaaagcagcagcagcagttgtccttcaggctgaaagggagttgcagccttggatcgccaaagatgatgaagaaggccaaaaaatgtggaaaatcaaccaacggatagtcaaagtgttggtggaactcatgcgcaatcatgacaggcctgagtcactggtgattctcgcaagtgcatcagatcttcttctgcgggcaactgatggaatgcttgttgatggagaagcttgtacattacctcaacttgagctacttgaagccacggcaagagcaatacagccggtgctagcttgggggccatctggactagcagtggtcgacggtttatccaatctattgaagtgtcgtctaccagcaacaatacggtgcctttcacacccaagtgcacacgtacgtgccttaagcacgtcagtactacgtgatatcatgaaccaaagctccatacccatcaaagtaactccaaaactgccaacaacagagaagaacggaatgaatagtccgtcctatcgattcttcaacgccgcctcaatagactggaaagccgatatccaaaactgtttaaactgggaagctcacagcttgctctccacaactatgcctactcagtttctcgacactgcggctcgggaactcggctgtactatatccttgtcccaagcggccgcaagtgctggtaagctactgtcttctatcgaacaagcatgcgatatttgccgacttaaaaagctcaagtgctccaaagaaaaaccgaagtgcgccaagtgtctgaagaacaactgggagtgtcgctactctcccaaaaccaaaaggtctccgctgactagggcacatctgacagaagtggaatcaaggctagaaagactggaacagctatttctactgatttttcctcgagaagaccttgacatgattttgaaaatggattctttacaggatataaaagcattgttaacaggattatttgtacaagataatgtgaataaagatgccgtcacagatagattggcttcagtggagactgatatgcctctaacattgagacagcatagaataagtgcgacatcatcatcggaagagagtagtaacaaaggtcaaagacagttgactgtatcgccgtaa

5. P_CMV_-LOV-VP16 (Addgene #42499)

6. P_UAS_-Luc (E137A, Promega)

7. P_UAS_-pre-pro-Insulin

The P_UAS_-Insulin is constructed through replacement of Luc from P_UAS_-Luc to Insulin (CCDS_7729.1).

Sequence:

cggagtactgtcctccgagcggagtactgtcctccgactcgagcggagtactgtcctccgagcggagtactgtcctccgagcggagtactgtcctccgagcggagtactgtcctccgagcggagtactgtcctccgagcggagtactgtcctccgaggaattccggagtactgtcctccgaagacgctagcggggggctataaaagggggtgggggcgttcgtcctcactctagatctgcgatctaagtaagcttatggccctgtggatgcgcctcctgcccctgctggcgctgctggccctctggggacctgacccagccgcagcctttgtgaaccaacacctgtgcggctcacacctggtggaagctctctacctagtgtgcggggaacgaggcttcttctacacacccaagacccgccgggaggcagaggacctgcaggtggggcaggtggagctgggcgggggccctggtgcaggcagcctgcagcccttggccctggaggggtccctgcagaagcgtggcattgtggaacaatgctgtaccagcatctgctccctctaccagctggagaactactgcaactagggctacttaccaaagaaagaaagag
